# Influence of CrossFit and Deep End Fitness training on mental health and coping in athletes

**DOI:** 10.3389/fspor.2023.1061492

**Published:** 2023-10-02

**Authors:** Rachel Cansler, Jerome Heidrich, Ali Whiting, Don Tran, Prime Hall, William J. Tyler

**Affiliations:** ^1^ Department of Psychology, New York University, New York, NY, United States; ^2^ Deep End Fitness, San Diego, CA, United States; ^3^ IST, LLC, Birmingham, AL, United States

**Keywords:** CrossFit, mental health, aquatic training, stress, athlete, brain health and performance

## Abstract

Physical exercise is known to improve mental health. Athletes can experience unique physical and emotional stressors, which can deteriorate mental health and cognitive function. Training apathy can lead to cognitive dissonance and further degrade performance by promoting maladaptive, avoidance coping strategies. Introduction of psychosocial and training variables, such as those used in CrossFit (CF) and other community-based fitness programs with strong peer support have been shown to help reduce training apathy and negative affect. Here, we explored whether addition of psychophysiological variation, experienced as “hunger for air” during underwater breath-hold exercises, could provide unique mental health benefits for athletes. We studied the influence of CF and Deep End Fitness (DEF), a community-based underwater fitness program, on several outcome measures of mental health and emotional well-being in volunteer athletes. We observed a significant reduction in stress scores of both the control CF training group and the experimental DEF group. We found that DEF produced a significant improvement in positive affect while CF training did not. Further supportive of our hypothesis that the psychological and biological stressors experienced in underwater, breath-hold training cause positive adaptive changes and benefits, DEF training uniquely increased problem-based coping. While our observations demonstrate both CF and DEF training can improve mental health in athletes, DEF produced additional, unique benefits to positive coping and attitudes of athletes. Future studies should further evaluate the broader benefits of community-based, underwater training programs on psychological and physiological health in athletes and the public.

## Introduction

Given the prevalence of those affected by mental health disorders (970 million worldwide) and reported increased rates of mental health-related issues since the COVID-19 pandemic, it should come as no surprise that our awareness of and attention to individual mental health has led to an upsurge in lifestyle management solutions in recent years (1). Of the lifestyle approaches available, physical activity and exercise are backed by a substantial amount of evidence that supports their benefits for depression, anxiety, psychological distress, and overall mood (1–3). Paradoxically, the pressure athletes experience in training and competitions can evoke a myriad of mental health issues including increased stress, anxiety, and depression (4). Hammond et al. (5) found the prevalence of depression among elite athletes (34%–68%) to be significantly higher than that reported in the general population (17% for adults aged 19–34) or intercollegiate athletes [21% (5);]. These mental health issues can deplete athletic performance, reduce the likelihood of competition success, and have a negative effect on an athlete's quality of life and general well-being.

Successful athletes must focus on psychological training that incorporates motivational techniques, goal setting, cognitive and emotional management, and mental focus training to obtain optimal performance (6, 7). It has been suggested that mental resilience training can help athletes mitigate their stress, anxiety, and depressive symptoms as well as optimize performance in sport and life (8). Building mental resilience (or mental toughness) helps improve self-confidence (i.e., one's sense of control) and mental stability, such that they remain relatively impervious to competition or hardship (8, 9). Athletes with a high level of mental toughness, more effectively manage training and competition demands while maintaining confidence and control under pressure (8, 9). In general, healthy coping is required to appropriately deal with stress, anxiety, and adversity (10). Consistent with the idea that athletes should incorporate psychological training alongside their physical training, these results underline the importance of including mental resilience methods and positive coping strategies in athletic training.

Some psychological and social aspects of physically intense, community-based programs like CrossFit have been shown to produce improvements in mental functioning amongst athletes (11–13). The introduction of psychological stress and other physiological variables to training paradigms like those experienced during breath-hold diving and training (14) may produce additional improvements in the mental health of athletes. By incorporating unique stress experienced underwater during breath-holds, Deep End Fitness (DEF) was designed to improve athletes' ability to employ top-down cognitive control techniques over emotional and physiological responses to stressful situations (15). This is achieved through a multi-modal training approach applied in inclusive, community workout sessions at public and private aquatic centers. Training includes breathwork exercises stressing nasal breathing and diaphragmatic breathing leading into breath-holds during land-based exercises before entering the water. The benefits of deep, diaphragmatic (or meditative) breathing are well-established and have been shown to reduce stress while improving heart rate variability, alpha brainwave activity, and top-down emotional control (16). Upon entering the water, training includes treading, static breath-holds, bobbing exercises, and breath-holds during underwater exercises that elicit psychological and physiological stress (fear) by producing “hunger for air” (17, 18). We hypothesize that teaching athletes to focus and accomplish tasks under this reflexive stress can translate to improved mental health and cognitive functioning. To begin testing this hypothesis, in the present study we investigated the influence of CrossFit and DEF training on mental health and coping outcomes in a group of multi-sport athletes.

## Materials and methods

### Study participants

Prior to study enrollment, participants were screened and provided informed consent. Participants filled out demographic surveys to assess their age, gender, and experience level. All study procedures and protocols were approved by the Solutions IRB (Yarnell, AZ). Healthy adult athletes already engaged in at least 180 min of vigorous physical exercise per week were recruited across multiple sports (i.e., triathlon, mixed martial arts, rugby, running, cycling, swimming, baseball, American football, surfing, tactical athletes, and others). Athletes were assigned to a CrossFit (CF) control group or the experimental Deep End Fitness (DEF) group. The DEF participant pool (*N* = 48) consisted of 11 females (age = 29.9 ± 1.93 years) and 37 males (age = 34.3 ± 1.5 years) and the CF participants (*N* = 18) consisted of 13 females (age = 43.0 ± 2.90 years) and 5 males (age = 38.6 ± 2.60 years). Participants had diverse backgrounds and broad competitive experience (15.7% beginners, 64.3% amateur, 8.5% Olympic/collegiate level, and 11.5% professional athletes) and fitness levels (23.9% average, 61.9% above average, and 14.2% elite).

### Outcome measures

Following informed consent and study enrollment, all volunteer participants completed three scales designed to measure mental health functioning. The mental health outcome scales used were the Depression, Anxiety, Stress Scale [DASS-42; (19)], the Positive and Negative Affect Schedule [PANAS; (20)], and Brief-COPE (21, 22). Following completion of the study period, these scales were readministered to participants for comparison against baseline measures in a within-subjects manner.

### CrossFit and Deep End Fitness training

**Figure 1:**
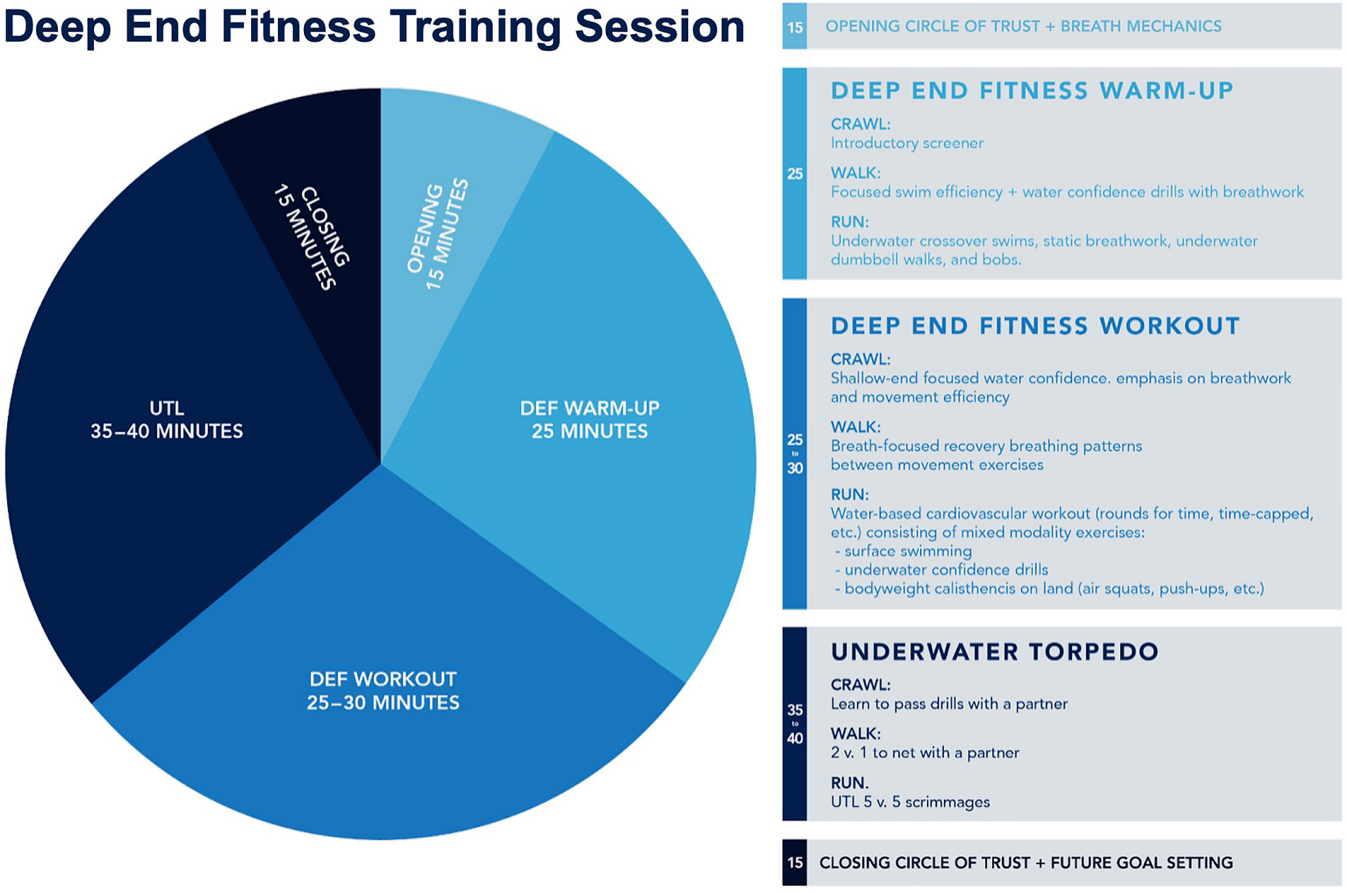
Overview of Deep End Fitness training sessions. A typical Deep End Fitness (DEF) training session lasts for 90 min. DEF training sessions begin and end with goal setting and performance review sessions conducted in a group manner (i.e., the Circle of Trust). DEF training sessions include stretching, land-based warm-ups, breathwork (i.e., diaphragmatic box-breathing, static breath-holds, dynamic breath-holds, etc.), and underwater physical exercises during.

**Figure 2:**
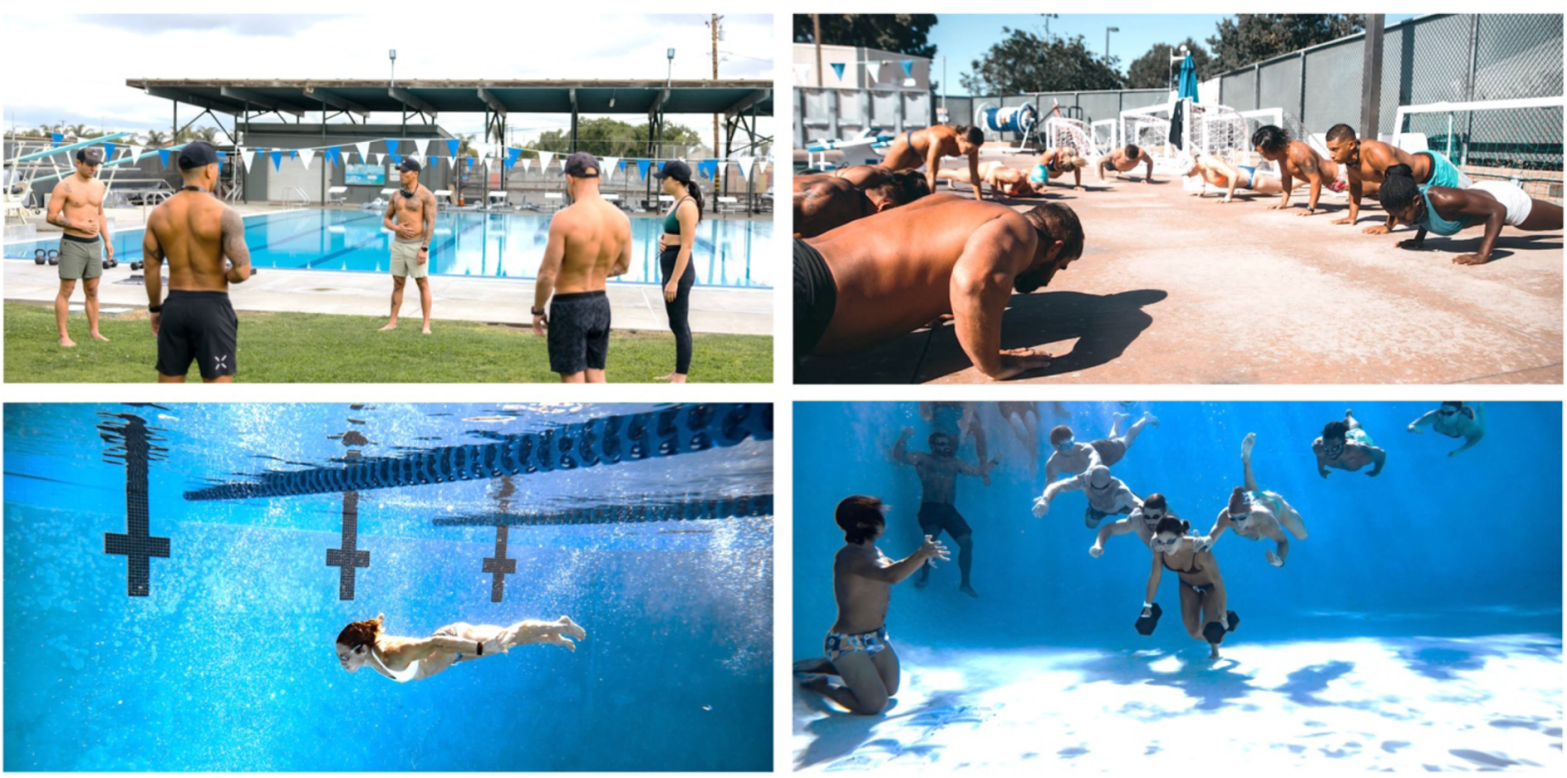
Community-based approach to Deep End Fitness training. Photographs illustrating the opening circle of trust with warm-up breath work (top-left) and a terrestrial warm-up that includes physical exertion during breath holding (top-right). Under supervised training with safety divers, DEF training includes underwater physical training regimens like 25-meter submerged swims (bottom-left) and underwater dumbbell carries (bottom-right) during dynamic breath-holds.

Participants were asked to attend at least one CF or DEF training session per week over a one to two-month period. Attendance to a minimum of three CF or DEF training sessions over a four-week period was required for inclusion in the study. CF training sessions consisted of standard routines including a warm-up, strength training period, high-intensity training period, and a cool-down period. DEF training session consisted of dynamic stretching, breathwork (e.g., diaphragmatic box breathing), mental focus training, mobility, and strength exercises on land and underwater during breath-holds (see Figures 1, 2). As part of routine safety practices, DEF training sessions were supervised by at least two certified DEF instructors, a lifeguard, and safety divers. All participants were required to practice safe buddy diving practices during training sessions.

### Statistical analyses

We used a mixed experimental design with between- and within-subjects analyses. We used ANCOVAs with competition and fitness levels as covariates to evaluate group main effects between CF and DEF training groups. We used post-hoc *t*-tests with Bonferroni's correction for multiple comparisons to analyze within-subjects differences between baseline and post training outcomes measures. Data were analyzed using JASP (JASP 0.16; University of Amsterdam, Netherlands) and R (GNU General Public License). All data are shown as Mean ± SEM. A *P-*value <0.05 was considered statistically significant.

## Results

### CrossFit and Deep End Fitness training improve mental health outcomes in athletes

**Figure 3:**
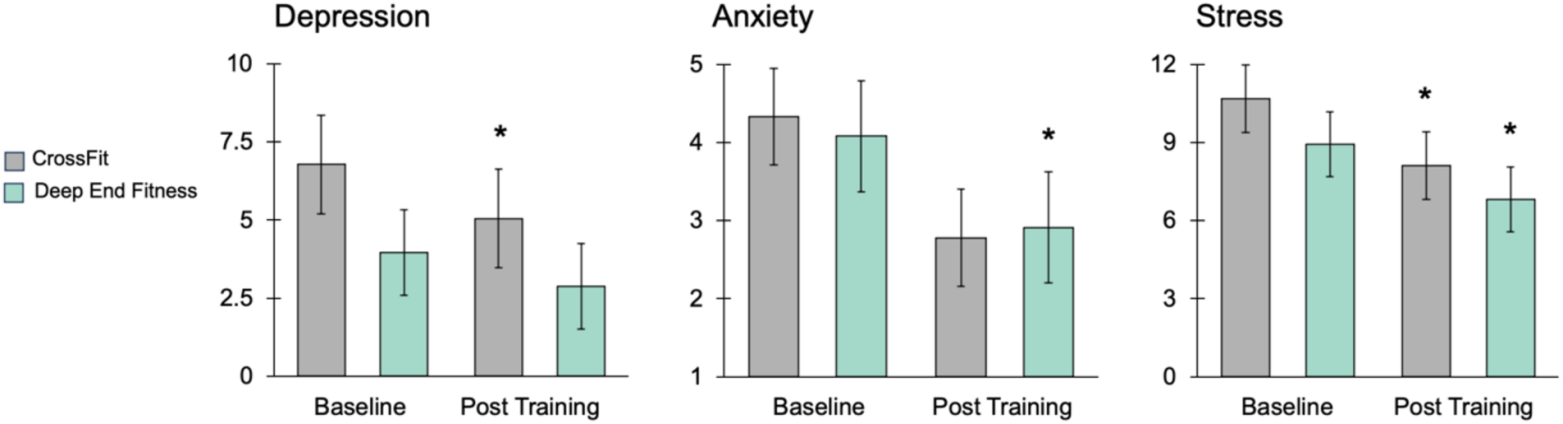
Influence of CrossFit and Deep End Fitness on depression, anxiety, and stress. Histograms illustrating the results produced by CrossFit (CF) and Deep End Fitness (DEF) training on depression, anxiety, and stress scores. The data illustrate CF and DEF training produce similar mental health benefits. Specifically, CF training produced a significant decrease in depression, DEF produced a significant decrease in anxiety, and both CF and DEF produced significant reductions in stress scores. An asterisk indicates *P* < 0.05.

Measurements of participants' depression, stress, and anxiety were assessed using the Depression, Anxiety, and Stress Scale [DASS; (19)] administered before and after the CrossFit (CF) control and experimental Deep End Fitness (DEF) training period. An ANCOVA on baseline and post-training depression scores revealed there was no significant group main effect between CF and DEF [*F* (1, 62) = 1.01, *P* = 0.32; Figure 3]. Post-hoc analyses of within-subjects data showed however that CF training produced a significant reduction in depression scores (baseline = 6.78 ± 1.58, post-CF = 5.05 ± 1.37, *P* = 0.003; Figure 3). An ANCOVA on baseline and post-training anxiety scores showed there was no significant group main effect between CF and DEF [*F* (1, 62) = 0.005, *P* = 0.94]. Post-hoc analyses revealed that DEF produced a significant 28.6% reduction in anxiety scores (baseline = 4.08 ± 0.52, post-DEF = 2.91 ± 0.44, *P* = 0.01; Figure 3). An ANCOVA on baseline and post-training stress scores showed CF and DEF produced similar effects to one another since there was no significant group main effect [*F* (1, 62) = 0.06, *P* = 0.81]. Post-hoc analyses revealed that CF produced a significant 23.9% reduction (baseline = 10.67 ± 1.30, post-CF = 8.11 ± 1.24, *P* = 0.016) in stress scores while DEF produced a similar significant 23.7% reduction (baseline = 8.93 ± 0.78, post-DEF = 6.81 ± 0.71, *P* = 0.007; Figure 3). Collectively, these results indicate that CF and DEF training produce similar, positive impacts on mental health as measured by depression, anxiety, and stress scores in athletes.

### Influence of CrossFit and Deep End Fitness training on affect

**Figure 4:**
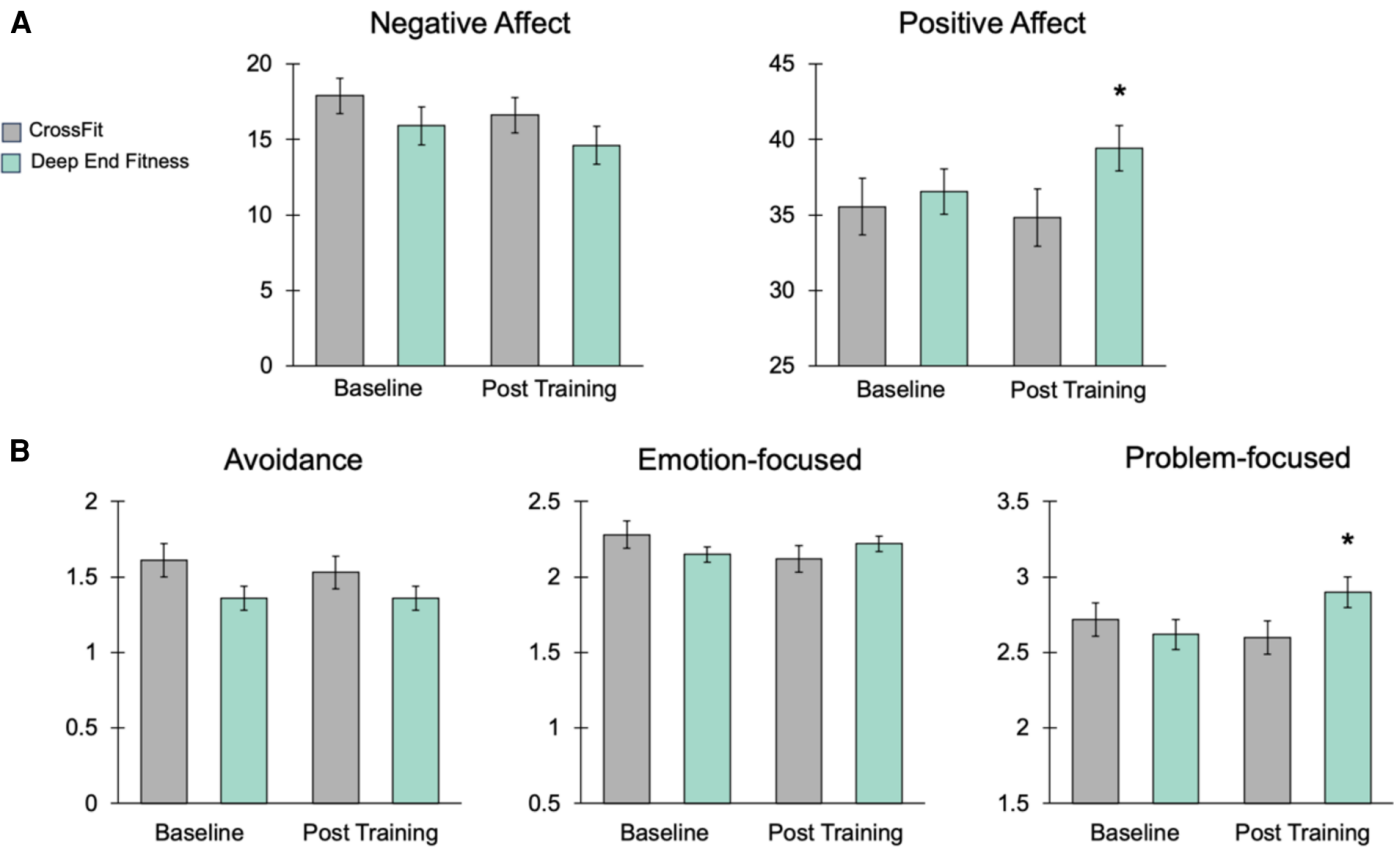
Deep End Fitness increases positive affect and problem-focused coping strategies. (**A**) Histograms illustrating effects of CF and DEF training on PANAS scores. The data show a significant increase in positive affect produced by DEF. (**B**) Histograms illustrating the effects of CF and DEF training on scores from the Brief-COPE survey. The data show DEF produced a significant increase in the use of problem-focused coping approaches. An asterisk indicates *P* < 0.05.

We measured positive and negative affect in volunteer athletes before and after CF and DEF training periods using the Positive Affect Negative Affect Scale [PANAS (20);]. An ANCOVA on baseline and post-training negative affect scores revealed no significant group main effect between CF and DEF [*F* (1, 62) = 0.002, *P* = 0.96]. However, there was a significant group main effect between CF and DEF training on positive affect scores [ANCOVA *F* (1, 62) = 3.98, *P* = 0.05; Figure 4A]. DEF produced a significant increase in positive affect (baseline = 36.5 ± 0.93, post-CF = 39.2 ± 0.92, *P* = 0.006; Figure 4A). These observations indicate that DEF training can positively affect mental attitudes in manner different than CF training.

### DEF training increases problem-based coping strategies

We also explored the effects of CF and DEF training on coping methods and strategies using the Brief-COPE survey (22). The BRIEF-cope is a 28-item, clinically validated scale assessing three coping strategies including avoidance, emotion-focused, and problem-focused. Avoidance and emotion-centered coping are indicative of poor mental health functioning, while problem-focused coping reflects healthy and positive mental approaches to dealing with stress (10, 21–23). An ANCOVA on baseline and post-training avoidance coping scores revealed no significant group main effect between CF and DEF [*F* (1, 62) = 0.46, *P* = 0.50]. Analysis of baseline and post-training emotional-based coping scores revealed a marginally significant group main effect [ANCOVA *F* (1, 62) = 3.92, *P* = 0.052], but post-hoc analyses failed to identify any within-group differences (Figure 4B). Analysis of baseline and post-training problem-based coping scores revealed a significant group main effect [ANCOVA *F* (1, 62) = 4.46, *P* = 0.04; Figure 4B]. Further post-hoc analyses showed DEF produced a significant increase in the use of problem-based coping strategies by athletes (baseline = 2.62 ± 0.10, post-DEF = 2.90 ± 0.11, *P* = 0.02; Figure 4B). Collectively, these data demonstrate DEF training can improve positive attitude and healthy, problem-focused coping approaches.

## Discussion

In this study, we examined the psychological influence of a novel underwater fitness training paradigm by comparing the effects of CrossFit (CF) and Deep End Fitness (DEF) training on mental health outcomes in athletes. In general, we observed that both CF and DEF training produced positive mental health benefits. Our observations indicate however, that exposure to the unique psychophysiological stressors experienced when underwater, breath-holding during DEF training may be responsible for specific improvements in positive affect and increased problem-based coping compared to CF. The community-based nature of CF and DEF training likely contributed to some of the improvements we observed. In fact, community-based or group fitness programs have been shown to foster a sense of belonging and foster other health benefits (24–27). The sense of community has been shown to be one of the most appreciated benefits of individuals engaged in CF training (11). In other work, we have found that more than 70% of athletes participating in DEF training rank the sense of community as one of their favorite aspects of the workouts (data not shown).

We found that DEF training significantly reduced anxiety and stress in athletes, as well as improved positive affect and problem-focused coping. The “hunger for air” and physical and psychological stress experienced during DEF training presents athletes with unique mental and physical challenges (17, 18). DEF training involves the teaching of cognitive control, stress reduction, and focusing techniques that help athletes overcome some of the mental and emotional difficulties of breath-hold diving. Encouraging athletes to set goals for achieving underwater physical challenges and equipping them with a skillset to achieve these goals, builds confidence and reinforces problem-focused coping as indicated by our data (Figure 4B).

Other physiological aspects of submerged, breath-hold diving may have contributed to the specific improvements in coping strategies we observed following DEF training compared to CF. As mentioned above, DEF training facilitates the learning of strategies for enduring psychological and physical stress that require participants to remain focused and goal-oriented when completing submerged exercises. Underwater, an athlete's mammalian diving reflex (DR) is initiated through stimulation of the trigeminal and vagus nerves to produce bradycardia, vasoconstriction, and slow oxygen metabolism (28–32). Breath-hold diving and underwater immersion, as methods of natural vagus nerve stimulation, encourage physiological relaxation and perhaps reinforces learning. Recently, multiple lines of evidence demonstrate that pairing brief periods (seconds to minutes) of non-invasive electrical vagus nerve stimulation with cognitive or physical tasks enhances brain plasticity, as well as training and learning outcomes (33–36). This poses the intriguing possibility that natural, vagus nerve stimulation experienced during DEF training reinforces the use of certain cognitive strategies leading to the significant improvements in problem-focused coping we observed (Figure 4B). Other lines of evidence show that when conducted with proper training, supervision, and safety support, breath holding can produce distinct neurophysiological states, which can benefit performance under stressful conditions (14, 37).

Strict safety protocols and supervised training are required to engage in any aquatic activity especially breath-hold diving. Blackouts, narcosis, barotrauma, decompression sickness, drowning, and other pulmonary trauma are known adverse events to occur amongst deep free breathing divers (30, 38–41). Improper training, poor safety preparation, inadequate supervision, and other environmental factors are leading causes of these traumatic injuries, accidents, and adverse events (41–43). DEF training sessions are always conducted with at least two certified instructors and under the supervision of a lifeguard and safety divers. Every training session begins with a safety briefing and all participants must use buddy diving practices. The depth and length of time breath-hold divers go to also causes complications. DEF training occurs in pools with average depths of four to six meters and breath-holds typically do not exceed two minutes during workouts. Hyperventilation or “air-packing” techniques are not allowed during DEF training as these poor practices are known to increase hypercapnia and blackouts. We observed no blackouts during our study. Independently, we are working with Red Cross to improve education, regulations, and procedures to enhance drowning prevention and preparedness. Between 19% and 38% of individuals participating in CF have experienced an injury during training (44–46). These injuries are similar across a variety of other high impact sports. We propose the low-impact nature of DEF training, even during submerged breath-hold physical exertion, provides athletes with a safe and unique alternative to other high-impact, community-based training programs prone to injuries.

There are several limitations to our study and observations. First, we only explored the impact of CF and DEF training across multiple weeks. Future studies are required to explore the chronic influence of DEF training across month long time scales. Second, we examined the influence of CF and DF in a limited, but diverse group of conditioned athletes. It is worth pursuing studies of DEF in larger cohorts of specific athletes to determine if some sport disciplines are affected more than others. Similarly, future investigations are required to study the impact of DEF training on individuals, who are not competitively active in sports. Additional limitations of our study are imposed by the difficulty of matching physical exertion levels across CF and DEF training conditions. While DEF includes moderately intense, interval exercises underwater, CF includes high-intensity interval training on land. It is difficult therefore to draw more specific conclusions about differences between CF and DEF at this point. It will be important to treat physical exertion and workload as variables to truly understand how DEF differs from other training regimens. Investigations into the impacts of DEF on cardiometabolic activity are also important to pursue. Despite the limitations of our study, the data do provide initial evidence that DEF can produce positive benefits for athletes.

Although the athletes in the present study did not suffer from any diagnosed mental health disorders, we are planning studies to evaluate the impact of DEF training on athletes diagnosed with generalized anxiety disorder, post-traumatic stress disorder, and depression. These studies represent a major growing area of interest due to the crucial need for new solutions to the mental health crises amongst competitive athletes and the broader public. As such, practical applications of DEF training may be experienced in employee team building exercises, confidence building, executive coaching, and mental resiliency training. In ongoing efforts, DEF training is being applied in this manner to improve water confidence and mental resiliency in professional athletes, tactical operators, and first responders. Indeed our data indicate that DEF training may positively impact mental health in populations beyond athletes, which warrants further investigations.

## Data Availability

The raw data supporting the conclusions of this article will be made available by the authors, without undue reservation.
